# Identifying the chloroperoxyl radical in acidified sodium chlorite solution

**DOI:** 10.1371/journal.pone.0252079

**Published:** 2021-05-26

**Authors:** Hiroyuki Kawata, Masahiro Kohno, Kohei Nukina, Isanori Horiuchi, Hisataka Goda, Tomomi Kuwahara, Kosei Yoshimori, Akimitsu Miyaji, Toshiaki Kamachi, Toshikazu Yoshikawa

**Affiliations:** 1 Sankei Co. Ltd., Shiromi, Chuou-Ku, Osaka, Japan; 2 Department of Life Science and Technology, Tokyo Institute of Technology, Ookayama, Meguro-ku, Tokyo, Japan; 3 Department of Microbiology, Faculty of Medicine, Kagawa University, Miki, Kagawa, Japan; 4 School of Materials and Chemical Technology, Tokyo Institute of Technology, Nagatsuta-cho, Midori-ku Yokohama, Japan; 5 Louis Pasteur Center for Medical Research, Tanaka Monzen-cho, Sakyo-ku, Kyoto, Japan; Beijing Foreign Studies University, CHINA

## Abstract

The present study identified the active radical species in acidic sodium chlorite and investigated the feasibility of quantifying these species with the diethylphenylenediamine (DPD) method. Electron spin resonance (ESR) spectroscopy was used to identify the active species generated in solutions containing sodium chlorite (NaClO_2_). The ESR signal was directly observed in an acidified sodium chlorite (ASC) aqueous solution at room temperature. This ESR signal was very long-lived, indicating that the radical was thermodynamically stable. The ESR parameters of this signal did not coincide with previously reported values of the chlorine radical (Cl^●^) or chlorine dioxide radical (O = Cl^●^-O and O = Cl-O^●^). We refer to this signal as being from the chloroperoxyl radical (Cl-O-O^●^). Quantum chemical calculations revealed that the optimal structure of the chloroperoxyl radical is much more thermodynamically stable than that of the chlorine dioxide radical. The UV-visible spectrum of the chloroperoxyl radical showed maximum absorbance at 354 nm. This absorbance had a linear relationship with the chloroperoxyl radical ESR signal intensity. Quantifying the free chlorine concentration by the DPD method also revealed a linear relationship with the maximum absorbance at 354 nm, which in turn showed a linear relationship with the chloroperoxyl radical ESR signal intensity. These linear relationships suggest that the DPD method can quantify chloroperoxyl radicals, which this study considers to be the active species in ASC aqueous solution.

## Introduction

xChlorine dioxide-based elemental chlorine-free (ECF) technology has long been the dominant process in the pulp and paper industry to produce bleached chemical pulps [1, 2]. Chlorine dioxide is usually generated when sodium chlorate or sodium chlorite in highly acidic conditions is reduced using hydrochloric acid, sulfuric acid, and hydrogen peroxide [2, 3]. In this process, chlorous acid (HClO_2_) is a reaction intermediate, and it has been shown to disproportionate when chlorine dioxide is generated under various conditions [2–5].

It is well known that in an acidic solution of NaClO_2_, ClO_2_ is formed by the following reactions [2, 4, 6]:

$${\text{NaClO}}_{2}+{\text{H}}^{+}\to {\text{HClO}}_{2}+{\text{Na}}^{+}$$ (1)

$$4{\text{HClO}}_{2}\to 2{\text{ClO}}_{2}+{\text{ClO}}_{3}{}^{-}+{\text{Cl}}^{-}+2{\text{H}}^{+}+{\text{H}}_{2}\text{O}$$ (2)

Eq (2) is the stoichiometry of the disproportionation of chlorous acid reported by several researchers [2, 4, 7–9]. This reaction under acidic conditions forms chlorate ions, chlorine dioxide, and chloride ions. Ni et al. [2] reported that chlorous acid disproportionation proceeded via stepwise reactions under strongly acidic conditions.

$${\text{2HClO}}_{2}\to {\text{HOCl}+\text{HClO}}_{3}$$ (3)

$${\text{ClO}}_{2}{}^{-}+{\text{HClO}}_{2}\to \text{HOCl}+{\text{ClO}}_{3}{}^{-}$$ (4)

$${\text{HOCl}+\text{ClO}}_{2}{}^{-}\to {\text{OH}}^{-}{+\text{Cl}-\text{ClO}}_{2}$$ (5)

$$\text{Cl}-{\text{ClO}}_{2}+{\text{HClO}}_{2}\to {\text{H}}^{+}+{\text{Cl}}^{-}+2{\text{ClO}}_{2}$$ (6)

In this process, the reaction is initiated by hypochlorous acid produced simultaneously from the disproportionation of one or two molecules of chlorous acid with one chlorite ion. Subsequently, hypochlorous acid reacts with chlorite to form Cl-ClO_2_, an intermediate of dichlorine dioxide [7]. Dichlorine dioxide then reacts with chlorous acid to produce two molecules of chlorine dioxide [2].

ASC solution has recently received much attention as a disinfectant in the food industry [10–17]. There are several studies that clarify the microbicidal and sterilization effect of ASC aqueous solution/chlorous acid [18–20]. There is no evidence, however, that points to which active molecules produce this microbicidal and sterilizing effect. Therefore, the purpose of this study was to use ESR spectroscopy to identify the main active species in an ASC aqueous solution and to quantify this species using the DPD method.

## Materials and methods

### Chemicals

Sodium thiosulfate, sodium sulfate anhydrate, sodium chlorite (purity: 80%), sodium hypochlorite, hydrochloric acid, sulfuric acid, potassium iodide, and *N*,*N*-diethyl-*p*-phenylenediamine (DPD) were purchased from FUJIFILM Wako Pure Chemical Co. Ltd. Starch was purchased from Kanto Chemical Co., Inc. 4-Hydroxy-2,2,6,6-tetramethylpiperidin-1-oxyl (TEMPOL) was purchased from Sigma-Aldrich.

### Preparations of ASC

An ASC aqueous solution was prepared by mixing 0.1 M sodium chlorite with 2.5 M hydrochloric acid using ion-exchanged water to the desired concentration previously mentioned. The concentration of sodium chlorite was measured by standard iodometric titration [18].

### Measurement of the free chlorine concentration

The free chlorine concentration was measured by the DPD method mentioned previously [18].

### Titration of ASC aqueous solution with thiosulfate

Active chlorine can be measured via thiosulfate titration [21]. ASC aqueous solutions were prepared by mixing a 1:1 ratio of 0.1 M sodium chlorite aqueous solution with 0.62 M hydrochloric acid for 20 minutes. Absorption at 354 nm was measured 10 min after adding sodium thiosulfate. The decrease in the average absorption at 354 nm was plotted against the final concentration of sodium thiosulfate in the ASC aqueous solution to clarify the relationship between the active chlorine and absorbance at 354 nm.

### Spectrophotometric analysis of ASC

UV-vis absorption spectra were recorded on a U-5100 ratio beam spectrophotometer (HITACHI). When the absorbance exceeded 1.5, the sample was appropriately diluted by ion-exchanged water. The absorbance of such samples was calculated by multiplying the dilution factor by the absorption at 354 nm.

### ESR analysis of ASC

ASC aqueous solution was placed into a glass capillary tube 100 mm long with a 2 mm inner diameter. The glass capillary was transferred into a capillary cell fitted in an ESR cavity, and ESR measurements were carried out at room temperature using an X-band spectrometer (JEOL FA-100ESR) operated at 9.43 GHz; the magnetic field was modulated at 100 kHz [22–24]. The conditions for measuring the ESR spectra were as follows: resonant frequency = 9.4599 GHz, microwave power = 4 mW, observed magnetic field = 336.0±10 mT, field modulation width of 0.1 mT, sweep time of 2 min, and time constant of 0.1 s. The signal intensities were normalized with respect to a MnO marker, and the concentrations of the stable radical products were determined based on the signal height using TEMPOL, an external standard. Radical species found in the ASC solution were quantified using integration software to determine the spectrum area mounted in the ESR device.

### Theoretical analysis

Models of the chloroperoxyl radical (Cl-O-O^●^) were prepared by drawing their molecular structure with GaussView 6.0, and the compound was optimized by density functional theory (DFT). We then considered the long-range and dispersion-corrected functional ωB97X-D, with the basis sets 6-311G(d,p) [25–27], in Gaussian 16 [28]. The model solvation effects were calculated with the conductor-like polarizable continuum model (CPCM); water was used as the solvent. The optimized molecular structures were verified through vibration analysis. We confirmed that the equilibrium structures did not have imaginary frequencies and that total energies were corrected at the zero-point vibrational energy.

## Results and discussion

### ESR analysis of radicals found in ASC

**Fig 1:**
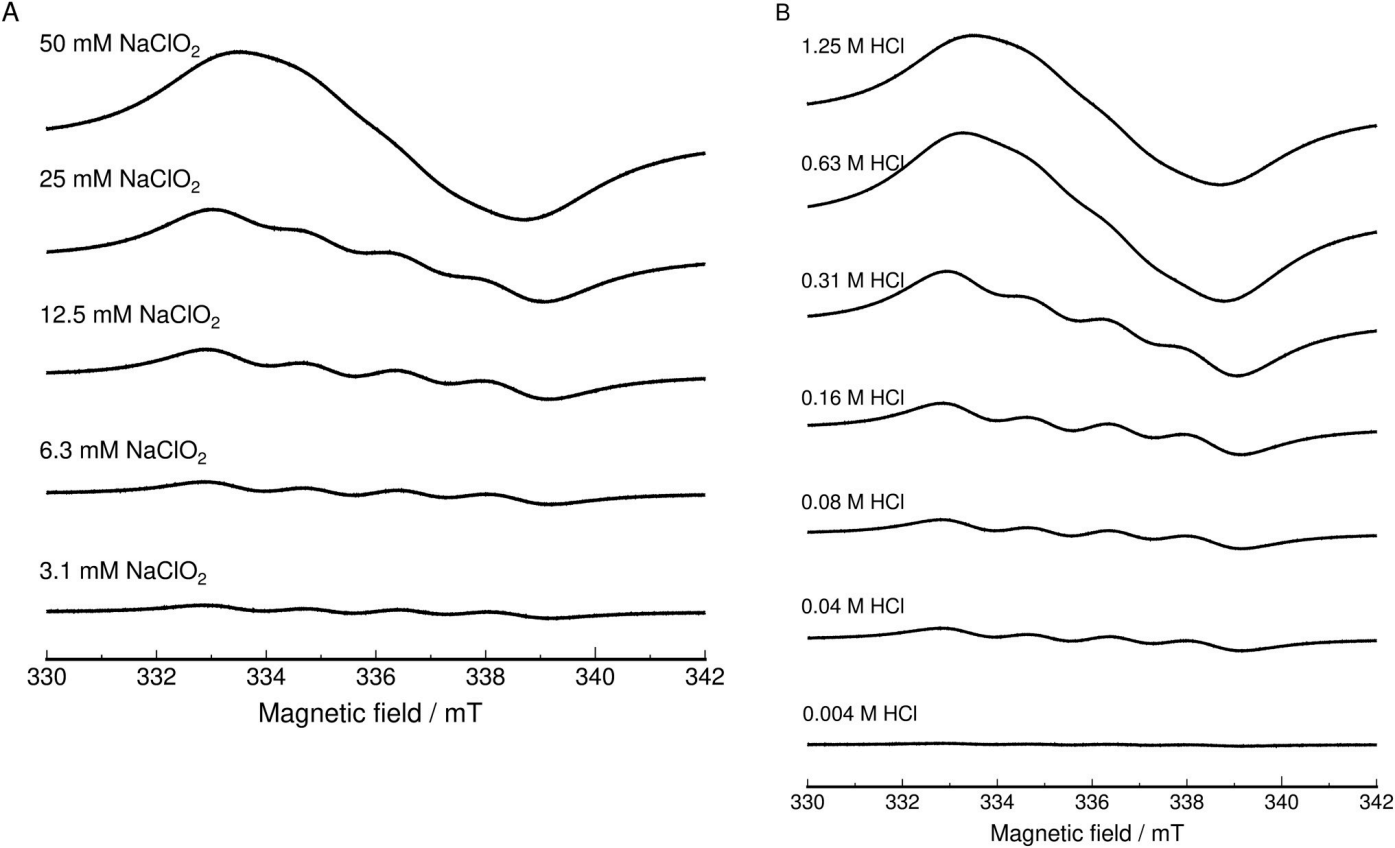
ESR spectra of radical species in ASC aqueous solution. (A) ESR spectra of radical species found in ASC aqueous solution after mixing with HCl aqueous solution to give a final HCl concentration of 1.25 M at 240 s. The concentration of sodium chlorite is indicated in the figure. (B) Dependence of ESR spectra of ASC aqueous solution on the HCl concentration. The final HCl concentration is indicated in the figure. The ESR spectrum was recorded 240 s after mixing 0.1 M sodium chlorite aqueous solution and HCl aqueous solution.

Free radicals in ASC aqueous solution were measured using ESR measurement. Sodium chlorite aqueous solutions with concentrations varying from 0.1 M to 0.0063 M were mixed with 2.5 M HCl aqueous solution at a ratio of 1:1. The solution was measured by ESR spectroscopy after 4 minutes. Fig 1A shows the ESR spectra of the ASC solution at room temperature. ESR signals found in Fig 1A are originated from one radical species supported by the same g-value (*g* = 2.0114 and α = 1.85 mT) found in both high and low concentration of NaClO_2_. Free radicals accounted for up to 30% of the chlorite concentration. Surprisingly, the solution showed a stable ESR signal under ambient conditions. We observed this ESR signal at least two weeks after preparation of the ASC solution. The lifetime of this ESR signal is very long, and the ESR signal intensities increased as the concentration of sodium chlorite increased. The broadening of the ESR signal found for high concentrations of NaClO_2_ and HCl is probably due to spin-spin interactions of radicals.

The ESR spectrum was split into four lines with an intensity ratio of 1:1:1:1. The ESR parameter values were *g* = 2.0114 and α = 1.85 mT, which coincide with data reported by Ozawa et al. [6, 29] (*g* = 2.0106, α = 1.85 mT). We calculated *I* = 1/2 and *S* = 3/2 from *E* = *hv* = *g*μв*H*_0_ + α*IS*. Thus, *S* = 3/2 indicates the chlorine isotopes 35Cl and 37Cl. Additionally, *g* = 2.0114 is the value calculated at the magnetic field (*H*_0_) = 336 mT from *g* = *hv*/μв*H*_0_. The magnetic field (*H*_0_) = 336 mT is the center of the four lines; two intensity ratios spread on both sides (totaling four ratios) of the magnetic field (*H*_0_) = 336 mT were observed (Fig 1A and 1B). Therefore, this signal pattern indicates two consecutive oxygen atoms to a chlorine atomic nucleus (Cl→O-O).

To examine the signal further, we compared our data with the ESR parameters of previously reported radical molecules. Hashimoto et al. [30] spin-trapped the superoxide anion (O_2_^●−^) in a strong alkaline DMSO solution with 5,5-dimethyl-1-pyrroline-N-oxide (DMPO) in a low-temperature solid state (77 K). The resulting ESR spectrum consisted only of a broad line with *g*_∥_ = 2.103 and *g*_⊥_ = 2.007, indicative of “O_2_^●−^” formation. The *g*_0_ value obtained for the isotropic superoxide solution was *g*0 = (*g*_∥_+2*g*_⊥_)/3 = 2.009 [30]. The *g*-value for the O_2_^●−^ adducts corresponded with that of the radicals found in the ASC aqueous solution in Fig 1. The ESR parameters reported by Adrian et al. [31] were *g*_z_ = 2.0100, *g*_x_ = 1.9987, and *g*_y_ = 1.9915 for “Cl-O-O” formation. This “Cl-O-O” structure was generated by similar photolysis of a sample consisting of 1% Cl_2_, 0.1% O_2_, and 98.8% Ar gas at 4 K [31]. The observed free radical was measured at cryogenic temperature, which suggests that it is a nuclear chlorine atom. Thus, the *g*_0_ value of the chlorine radical (Cl^●^) (or chlorine dioxide radical with a high electron density on the chlorine atom nucleus (O-Cl^●^-O)) gas was *g*_0_ = (*g*_z_+*g*_x_+*g*_y_)/3 = 2.000. This *g*_0_ value completely differs from the *g*-value reported in this paper. These results indicate that the “Cl-O-O” formation in Fig 1A may have a molecular structure similar to that of superoxide.

In the case of Eachus et al. [32], the ESR parameters reported were *g*_x_ = 1.9983 and α_x_ = 0.53 mT, *g*_y_ = 2.0017 and α_y_ = 0.72 mT, and *g*_z_ = 2.0130 and α_z_ = 1.49 mT for “chlorine peroxide, Cl-O-O”. The *g*_0_ value was *g*_0_ = (*g*_z_+*g*_x_+*g*_y_)/3 = 2.0043 and α_0_ = (α_z_+α_x_+α_y_)/3 = 0.913 mT. This “chlorine peroxide” of ref.32 was generated from KClO_4_ by irradiating with γ-rays at 195 K; however, the reported value, *g*_0_ = 2.009, was similar to that of the superoxide anion (O_2_^●−^). As a result, we believe the value reported by Eachus et al. [32] is for an oxygen radical (O^●^). On the other hand, *g*_0_ = 2.0043 is close to *g*_0_ = 2.000, the value for a chlorine radical (Cl^●^), rather than *g* = 2.0114. Additionally, α_0_ = 0.913 mT does not correspond with α = 1.85 mT. The sample structure of Eachus et al. [32] may be a chlorine dioxide radical (O = Cl-O^●^) with a radical present on the oxygen atom.

Based on the above considerations, the quadrupole splitting found in Fig 1A should be assigned to the ESR signal of “Cl-O-O^●^” with an oxygen radical and not chlorine dioxide radical (O = Cl^●^-O) with a high electron density on the chlorine atom nucleus. Therefore, this radical active species should be referred to as “Cl-O-O^●^” or chloroperoxyl radical.

Fig 1B shows the ESR spectrum after mixing 0.1 M sodium chlorite aqueous solution with HCl aqueous solutions ranging from 2.5 M to 0.008 M at a ratio of 1:1. The ESR signals intensified with increasing HCl concentration. This result indicates that chloroperoxyl radicals were only formed under acidic conditions.

### Relationship between chloroperoxyl radical intensity and absorbance

**Fig 2:**
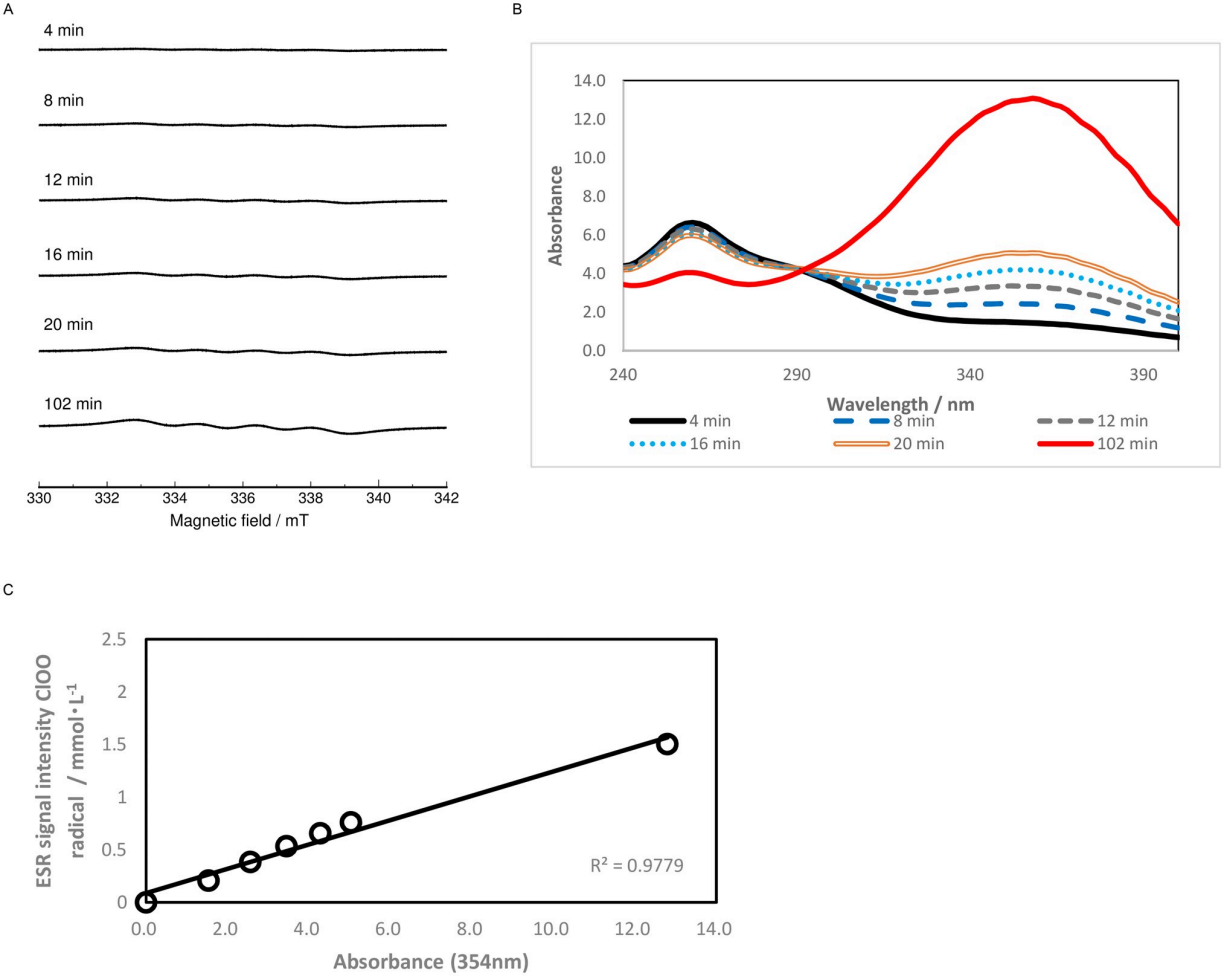
ESR signal intensities and UV-visible absorption spectra of ASC aqueous solution. (A) Time-dependent changes in ESR spectra after mixing 0.1 M sodium chlorite solution with 0.08 M hydrochloric acid at a ratio of 1:1. (B) Absorption spectrum of ASC aqueous solution under similar condition as in (A). (C) Relationship between the chloroperoxyl radical ESR signal intensities and absorbance at 354 nm obtained from (A) and (B).

As mentioned above, chloroperoxyl radicals form when sodium chlorite aqueous solution is acidified. Fig 2A shows the time-dependent changes in the chloroperoxyl radical signal after adding HCl. The chloroperoxyl radical ESR signal intensity gradually increased under relatively low concentrations of HCl, and this ESR signal remained stable for over a week when stored in the dark. Fig 2B shows the UV-vis absorption spectra of acidified ASC aqueous solution. Absorption spectra were measured under similar conditions as in Fig 2A. The absorption band at 354 nm also increased as the time after mixing increased. Fig 2C shows the relationship between the ESR signal intensity and absorbance at 354 nm obtained from Fig 2A and 2B. An apparent linear relationship exists between the chloroperoxyl radical signal intensity and absorbance at 354 nm. This result indicates that the absorption band at 354 nm usually reported with ASC solution originates from chloroperoxyl radicals.

**Fig 3:**
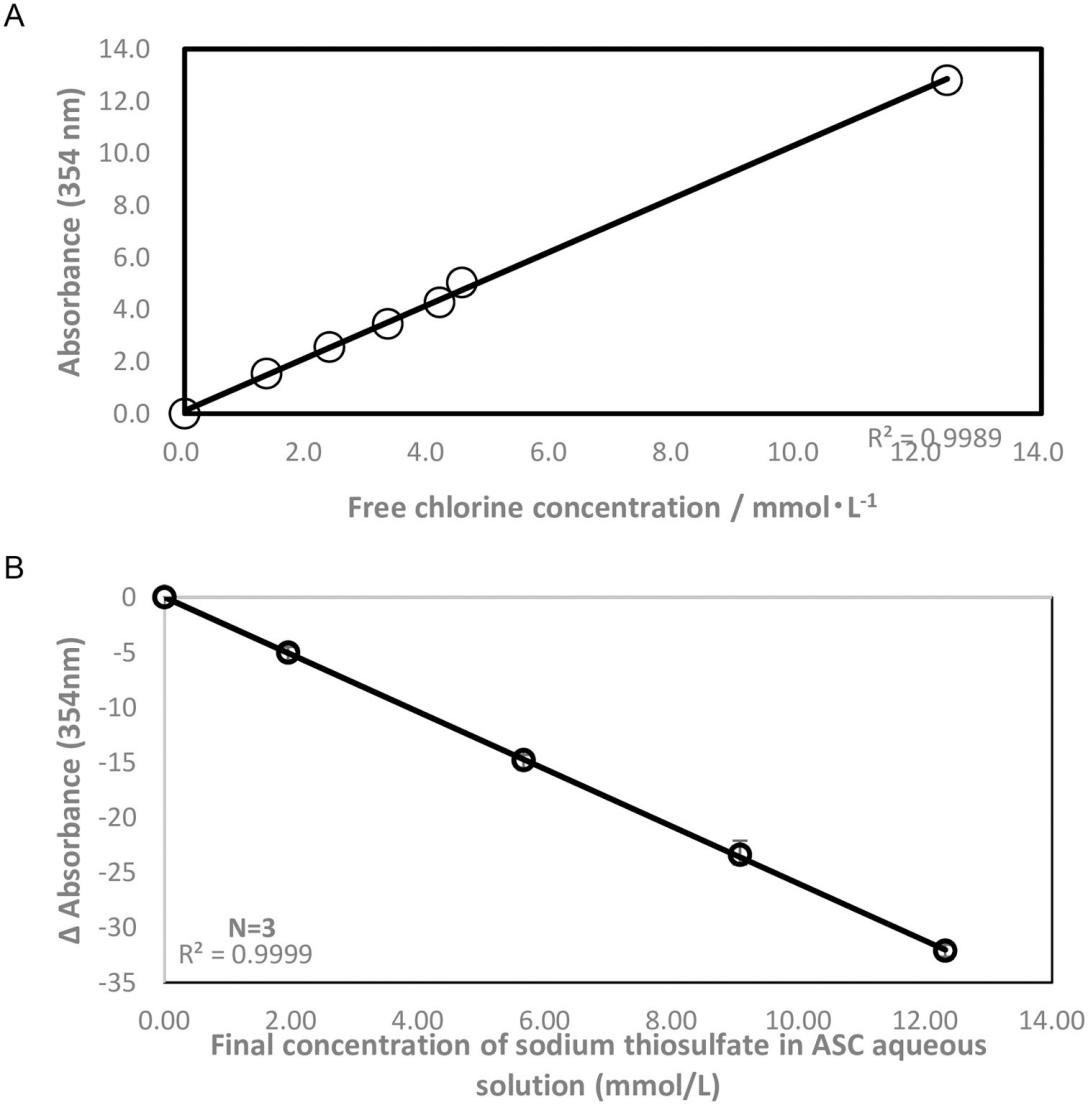
Relationship between A354 and active chlorine concentration in ASC solution and active chlorine consumption. (A) Relationship between the absorbance at 354 nm and free chlorine concentration obtained by the DPD method at the same reaction conditions as in Fig 2A. (B): Decrease in absorbance at 354 nm in ASC aqueous solution when thiosulfate was added. Data are the means ± SD (n = 3).

Fig 3A shows the relationship between the absorbance at 354 nm in the ASC aqueous solution and the free chlorine concentration after mixing sodium chlorite aqueous solution with HCl. As shown in Fig 2B, the absorbance at 354 nm increased in a time-dependent manner. The free chlorine (free, not combined and total chlorine) concentration was measured by the DPD method simultaneously with the UV-absorption spectrum. The free chlorine concentration also increased in a time-dependent manner and showed a good linear relationship with the absorbance at 354 nm, indicating a strong correlation between the two. Fig 3B shows the quenching experiment of free chlorine by adding thiosulfate. As the concentration of thiosulfate in the ASC solution increased, the absorbance at 354 nm decreased linearly, indicating that the absorbance at 354 nm is closely associated with active chlorine species. These data indicate that the absorbance at 354 nm originated from active chlorine, which reacted with thiosulfate; this active chlorine species was detected by DPD. In Fig 2, chloroperoxyl radicals show absorption maxima at 354 nm, which closely correlates with the active chlorine species that reacts with thiosulfate. This finding indicates the possibility that chloroperoxyl radicals are the active species in ASC solution. The absorbance at 354 nm was also long-lived. We measured the absorption band at approximately 354 nm three months after preparation of the ASC solutions.

The formation of this absorption band at approximately 354 nm originating from chloroperoxyl radicals did not change in the presence or absence of dioxygen molecules in the solution (S2 Fig in S1 File), suggesting that molecular oxygen is not involved in the formation of chloroperoxyl radicals.

It is well known that chlorite does not have antimicrobial effects. The total chlorine concentration includes both inactive species, such as chlorite, and active free radicals, such as chloroperoxyl radicals. Therefore, if the concentration of ASC aqueous solution is determined by standard iodometric titration, the concentration of active species in ASC aqueous solution will be overestimated despite not being enough to cause a sterilizing effect. As mentioned above, the DPD method and absorbance at 354 nm show a linear relationship with the radicals found in the ASC aqueous solution by ESR analysis, indicating that as an active free radical, chloroperoxyl in ASC aqueous solution can be quantified using DPD or absorbance measurements at 354 nm.

### The theoretical analysis of chloroperoxyl radical

Chloroperoxyl radical (Cl-O-O^●^) molecular models in a water phase were prepared by constructing their molecular structure by GaussView 6.0 and were optimized using DFT. The stability of chloroperoxyl radicals was quantified in an aqueous solution via a conductor-like polarizable continuum model (CPCM) with water as the solvent.

**Fig 4:**
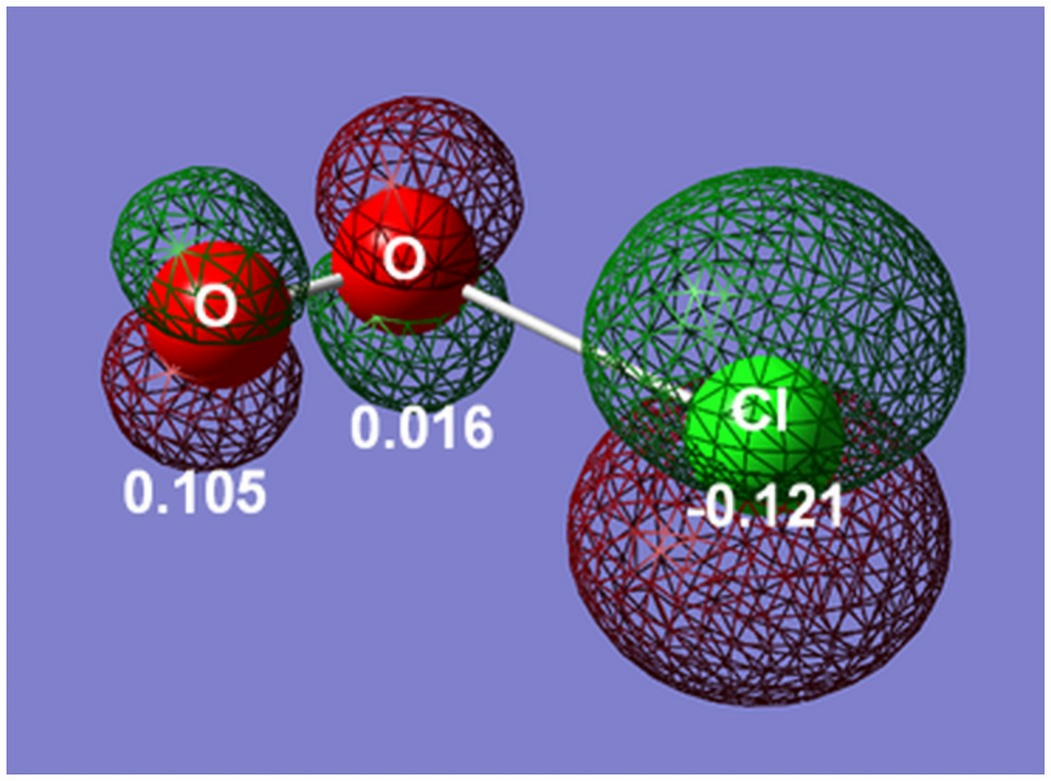
The optimized structure of chloroperoxyl radical and charge distribution and SOMO. The Cl atom and oxygen atoms are shown as green and red balls, respectively. The values show the Mulliken charge distribution on each atom, and the mesh surface shows the spin density of the SOMO.

The optimized structure of chloroperoxyl radicals and their singly occupied molecular orbital (SOMO) are shown in Fig 4. The bond length of Cl–O was 0.245 nm, while that of O–O was 0.119 nm. The O–O bond was shorter than that of molecular oxygen (O_2_, 0.121 nm) and was longer than or similar to that of dioxygenyl salts (O_2_^+^, 0.091–0.121 nm at room temperature) [33]. According to the Mulliken charge distribution shown in Fig 4, the negative charge was localized on the Cl atom. These structural properties suggest that the Cl atom of chloroperoxyl radicals is anionic, while the oxygen atoms are cationic like dioxygenyl cations.

The total energy of the optimized structure of chloroperoxyl radical (Cl-O-O^●^) and chlorine dioxide radical (O = Cl^●^-O), which are candidates for [ClO_2_]*, is shown in Table 1. The total energy of chloroperoxyl radical (Cl-O-O^●^) was 274 kJ/mol lower than that of chlorine dioxide radical (O = Cl^●^-O). This large difference indicates that the chloroperoxyl radical (Cl-O-O^●^) is more stable than the chlorine dioxide radical (O = Cl^●^-O) in a water phase. The theoretical result is consistent with the experimental observation that chloroperoxyl radicals (Cl-O-O^●^) showed a much longer lifespan than chlorine dioxide radicals (O = Cl^●^-O). Therefore, these results support the theory that the active radical species observed in the ESR measurement is the chloroperoxyl radical (Cl-O-O^●^).

**Table 1:** Total energy of Cl-O-O^●^ and O = Cl^●^-O calculated by DFT.

Radical species	Total energy (a.u.)	Relative energy (kJ/mol)
O = Cl^●^-O	-610.365367	0
Cl-O-O^●^	-610.469703	-274

## Conclusions

We measured free radicals in ASC aqueous solution by ESR spectroscopy and assigned chloroperoxyl (Cl-O-O^●^) as the radical present and not chlorine dioxide radical (O = Cl^●^-O). Total energy calculations by DFT showed that chloroperoxyl radicals are more stable than chlorine dioxide radicals. The active chloroperoxyl radicals in ASC aqueous solution can be quantified using the DPD method or absorbance measurement at 354 nm. The disinfectant ability of ASC is supposed to originate from the high concentration of chloroperoxyl radicals found in ASC solutions even though the stability of chloroperoxyl radicals is very high.

## Supporting information

S1 File (DOCX)
