# Supplementary material for: Identifying the chloroperoxyl radical in acidified sodium chlorite solution
Source: PLoS One. 2021 May 26;16(5):e0252079. doi: 10.1371/journal.pone.0252079 (PMC8153430; doi:10.1371/journal.pone.0252079)
Supplement: S1 File — (DOCX) [file pone.0252079.s001.docx]

**S1 File**

**S1 Fig. Spin-Trapping ESR Measurement of ASC Solution Using 1.5 M DMPO under Different Concentrations of HCl**. The NaClO_2_ concentration was 50 mM. The final concentration of HCl is indicated in the figure.

S1 Fig shows the ESR signal of the ASC solution obtained by spin-trapping with DMPO. The signal intensities of these signals were estimated by the comparison of standard TEMPOL solution and manganese oxide marker signals observed on both sides of the DMPO spin-trapping signal.

By the acidification of NaClO_2_ with the addition of HCl solution, the DMPO-X ESR signal intensities were increased, while the DMPO-OH signal originating from spin-trapping of hydroxyl radicals with DMPO was not detected. The observed DMPO-X signal was in the range of 5-8 µM.

**S2 Fig. Absorbance Change after the Addition of HCl Solution to NaClO_2_ Solution.**

Then, 100 mM NaClO_2_ and 12 M HCl were added to Milli-Q grade water to give 1 mM NaClO_2_ and 400 mM HCl. The absorbance was measured with a Shimadzu Multispec 1500 UV-visible absorption spectrometer. The ASC solution was continuously stirred during measurement. In the case of a nitrogen gas atmosphere, Milli-Q grade water was deaerated by bubbling it with nitrogen gas for 30 min before the experiment. After the addition of NaClO_2_ and HCl to aerated or deaerated Milli-Q water in a screw-top cuvette, the cuvette was closed with the screw cap and applied for measurement.

The formation of chloroperoxyl radicals measured by absorbance at 354 nm did not change in the presence or absence of oxygen. This observation indicates that chloroperoxyl radicals were not formed by the reaction of NaClO_2_-derived radical(s) and dissolved molecular oxygen in the solution. The mechanism of chloroperoxyl radical formation is unclear.
